# A recruiting failure turned success

**DOI:** 10.1186/1472-6963-8-64

**Published:** 2008-03-27

**Authors:** Alexis J Hure, Roger Smith, Clare E Collins

**Affiliations:** 1 Nutrition and Dietetics, School of Health Sciences, The University of Newcastle, Callaghan, NSW, Australia; 2 Mothers and Babies Research Centre, Hunter Medical Research Institute, John Hunter Hospital, New Lambton, NSW, Australia; 3 School of Medicine and Public Health, The University of Newcastle, Callaghan, NSW, Australia

## Abstract

**Background:**

This paper describes an attempt that was made to recruit child-bearing women into a nutrition-based research study and the knowledge that was gained when this approach was unsuccessful. The Assessment Before Children Develop Obesity Study was a cross-sectional survey which planned to follow-up women and children who had previously been, or were currently enrolled in the Mathematical Model of Pregnancy Study.

**Methods:**

Ethics approval was sought and obtained over an eight month period. After just six weeks it was obvious that our research objectives were not achievable because of an inadequate response rate (10%). This led to a review of the recruiting methodology as well as all written materials provided to potential participants. Advice was sought from those with expertise in the design of large public health campaigns and literature was consulted to refine our recruitment strategy.

**Results:**

In subsequent redevelopment, the Assessment Before Children Develop Obesity Study was merged with the Mathematical Model of Pregnancy Study to become what is now known as the Women and Their Children's Health Study. Consent rates improved from 10% and 35% in the Assessment Before Children Develop Obesity and Mathematical Model of Pregnancy studies respectively, to 61% in the Women and Their Children's Health Study (chi square test, p < 0.001). Successful recruitment for this research continues. The significant improvement in the participation rate is attributed to numerous factors including changes to the study name, recruiting method and information materials.

**Conclusion:**

By sharing our experience we aim to assist other researcher in avoiding the same pitfalls and offer effective strategies for improving response rates.

## Background

Recruiting is typically viewed as a means to an end, rather than a potential end in itself. This phase of the research project often turns out to be more of a challenge than anticipated. Recruiting difficulties can lead to: responder bias, an insufficient sample size to adequately power statistical analyses for hypothesis testing, costly delays in achieving the research objectives, and as a worst case, early cessation of the research project. There is limited literature to guide researchers in the practical aspects of recruiting for research studies. The magnitude of this problem has previously been demonstrated by Charlson and Horwitz (1984) who conducted a study into the impact of participant losses before randomisation. Using the multicentre trials listed in the 1979 inventory compiled by the National Institute of Health, they found that only 34% of trials ever reached their projected sample size [1].

In 2005 the Assessment Before Children Develop (ABCD) Obesity study was planned and developed as a PhD project at the University of Newcastle, Australia. The aim of this study was to investigate how maternal dietary intake affects the growth and development of the child, in both the pre and post-natal periods. To do this we had planned to utilise the established framework of another large cohort study which was already underway at the John Hunter Hospital, a tertiary referral centre and major obstetric facility for the Hunter region of New South Wales, Australia. The Mathematical Model of Pregnancy (Math Model) study had been established since 1999 to explore methods of predicting preterm birth [2]. It was identified as an appropriate database of eligible candidates from which to seek recruits for the ABCD Obesity study because of the significant overlap in the data that was to be collected. Further details of the Math Model study have previously been published elsewhere [2].

## Methods

### Participants

Research midwives were employed to approach potential recruits in the antenatal clinic of the John Hunter Hospital. All pregnant women who were up to 16 weeks gestation were eligible to participate in the Math Model of Pregnancy study. At this first encounter a brief verbal description of the study was provided and the recruiting materials were issues. Follow-up phone calls were usually made two or three days after first being approached, to determine willingness to participate. The same method of recruiting continued with the change over to the Women and Their Children's Health (WATCH) Study.

Just over 600 women who had previously participated in, or were currently enrolled in, the Math Model Study were to be invited to join the ABCD Obesity Study. It was estimated that with a response rate of 60 to 70%, between 360 and 430 mother-child (up to four years of age) pairs would be recruited into this study, in addition to 100 women who were currently pregnant. This estimate was thought to be conservative, given that these women had previously consented to research of a similar nature.

Due to the large sample size recruiting was to be staggered, with a sample of 50 women approached at a time. Potential participants were to be invited into the study from both ends of the Math Model timeframe, first approaching those who had been out of the study the longest (n = 25), as well as those who had most recently joined (n = 25). Of the 50 invitations to participate that were mailed out, a total of only five consents were received, even after a follow-up letter was distributed. Four of these consents came from women who were currently pregnant and were still actively involved in the Math Model study.

### Ethics Approval

Prior to submitting the ethics application for committee review, advice was sought from the Professional Research Ethics Officer on the appropriateness of re-recruiting subjects that had previously participated in research. Concerns were raised regarding the manner in which potential participants would be contacted, and measures (for example the mode of contact) were written into the research protocol to avoid consent out of a sense of obligation. The original application for the ABCD Obesity Study was made to the Hunter Area Research Ethics Committee in May 2005 and approval was obtained in September 2005. Prior to commencing recruitment, a variation to the original application was submitted and approval for this was received in October 2005. Recruitment then commenced immediately and continued for the duration of the next six weeks. After this time it was evident that we were not going to be successful in meeting our sample size targets and subsequently our research objectives, due to the very poor rate of response (10%) to the invitation to participate.

### Participant Involvement

Women who participated in the Math Model of Pregnancy Study consented to having three study ultrasound scans, at 18–20, 26 and 32 weeks gestation. At each of these study visits non-fasting blood and urine samples were collected. Umbilical cord blood was collected at birth. Participants had the option of donating one further blood sample while in labour and additionally, their placenta after delivery.

The information statement for the ABCD Obesity study documented the requirements of participation as attending two appointments at the John Hunter Hospital, at which physical measurements would be collected and questionnaires would be interview-administered for both mother and child. Dietary data recording for mother and child was a major component of the ABCD Obesity Study, including 3-day weighed food records and food frequency questionnaires. Maternal and child blood samples were also listed as optional components of the study.

The WATCH Study has combined the data that was planned for collection in both parent studies, using the prospective longitudinal study design that was already in place for the Math Model study, but with ongoing follow-up in infancy and early childhood. An extra ultrasound scan is performed at 36 weeks for the women in the WATCH Study who have not already delivered. Child follow-ups take place at 3, 6, 9, 12 and 24 months, with physical, medical and dietary data being collected at these study visits.

### Procedures

A review of the study design and all recruiting materials was undertaken in an effort to identify the shortcomings of the project. Evaluation and advice was sought from those with extensive expertise in recruiting, not only for research purposes but for large-scale public health interventions. Literature on recruiting participants was consulted [3-9]. All feedback was considered in the redevelopment of the project and a complete overhaul of the method of recruitment and the materials provided to potential participants took place.

## Results

One of the difficulties in evaluating what went wrong is that ethically we were not able to re-contact the women who did not consent to participate and ask them what factors contributed to their decision. We were able to ask those who did consent why they agreed to volunteer in further research. However this only highlighted that these women were likely to be different to the broader population we were targeting, with an enthusiasm and extreme willingness to be involved. We therefore relied upon the feedback sought from external sources (literature and expert consultation).

In doing so a number of issues were identified as potential contributors to the poor initial response rate. These are listed in Table 1 and are addressed in turn in the discussion. A complete overhaul of the study design and recruiting materials was considered necessary to both improve future recruitment and avoid unnecessary delays in our attempts to recruit successfully. Due to the extremely poor rate of response from the women who had previously participated in the Math Model study (one consent out of 25 invitations), we decided to concentrate our efforts on only women who would prospectively be recruited to the research project. The ABCD Obesity study was consequently amalgamated with the Math Model Study to become the WATCH Study. At this time both studies underwent an upgrade in the level of detail and attention that was devoted to the way in which potential participants were approached.

**Table 1:** Potential reasons for the recruiting failure of the ABCD Obesity study

**Study Design**	**Recruiting Materials**
The study name	Poor visual appeal
The selected participant group	Length of the information statement
The method of approaching participants	Readability of the text
Inequitable benefit gained by the research team compared to participants	General approach and content
Ethics approval too highly prioritised	

In doing so our rate of response improved from 10% for the ABCD Obesity cohort, and 35% in the Math Model cohort, to 61% for the combined WATCH Study (Table 2). A response rate of 35% for the Math Model cohort was considered (at that time) reasonable, as this was the first invitation to participate in research that was issued, with many women simply opting to decline. Approximately 10% of women who consented to participate in the Math Model Study withdrew prior to their study completion at the time of delivery. The absolute number of women recruited to the study also increased from four per month in the Math Model cohort to 14 per month for the WATCH cohort (figures averaged over the six months preceding and following the changes). One of the logical reasons for the improvement in participation rates was the changes that were made to the recruiting materials. Table 3 shows the dramatic differences in the readability statistics of the recruiting materials for each research study. Despite the complexity of merging the ABCD Obesity and Math Model studies, the materials provided for the WATCH study were drastically simplified.

**Table 2:** Recruiting response rates for the ABCD Obesity, Math Model and WATCH Study

	**ABCD Obesity**	**Math Model**	**WATCH Study**	**P-value**
Approached n	50	65	141	
Consented n (%)	5 (10)	42(35)	86 (61)	<0.0001
Rejected n (%)	0 (*)	23 (65)	55 (39)	

* The women who received the letter of invitation to participate in the ABCD Obesity had the option of declining the invitation using a reply-paid self addressed letter.

**Table 3:** Recruiting materials readability statistics for the ABCD Obesity, Math Model and WATCH Study

	**ABCD Obesity**	**Math Model**	**WATCH Study**
Number of words	2127	1439	859
Number of A4 pages	5	3	2
Words per sentence	19.1	17.9	12.8
Number of paragraphs	96	66	53
Passive sentences	26%	31%	17%
Flesch reading ease^a^	50.1	45	66.3
Flesch-kincaid grade level^b^	10.9	11.3	7.1

^a ^Text is rated on a 100-point scale. The higher the score, the easier it is to understand [16]

^b^Text is rated on a school grade level [16]

## Discussion

### The study name

Feedback highlighted that from the outset the title ABCD Obesity may have been a deterrent. Initially there was consensus among the research team that having a strong term like 'obesity' in the study name would attract interest, in a similar fashion to its common use in public and popular press. In hindsight however, it was likely to be the opposite. The social interpretation from consumer literature tends to be pejorative in nature, and the study title was likely to be particularly discouraging to women who were above their healthy weight range [10,11].

### The participant group

There are ethical considerations that need to be assessed before commencing research involving a population or group who are often targeted or who have previously participated in research. The National Statement on Ethical Conduct in Human Research [12] provides guidance on what is appropriate and emphasises the need to respect the rights of the participant to decline. In practise, this sensitivity translated into the way that we were able to approach potential participants, as further described below. The mode of contact proved to be so unsuccessful that during the study redesign we redirected the focus to the women who were currently pregnant and had not previously been approached.

### Approaching participants

Sending a letter of invitation through the mail was always going to be a less than ideal recruiting strategy for several reasons. Firstly, we could not be sure that the intended recipient actually received the invitation, unless of course we received a reply. Conversely, we could not be sure where the recipient did not receive the invitation as a result of a change of address, unless our mail-out was delivered 'return to sender' (only two were returned in this way). Registered mail would have been useful, however this option was not considered at the time of the mail out.

In our case we were working from hospital records which may or may not have contained the potential participant's most current postal address. To put this in context, between 1996 and 2001, 40.5% of people living in the Hunter region had changed their address [13]. While 28% of these remained living in the Hunter region, 12.5% had moved either elsewhere in NSW, interstate or overseas[13]. These figures do not take into consideration multiple changes in address that may have occurred. Lack of most current data may have been a major contributor to the very poor response rate in the sample of women who had completed their involvement in the Math Model project several years ago.

For those who did receive the information, from the outset the onus was on them to establish contact with the research team whether to agree to participate or to seek more information. A reply-paid, self-addressed envelope was provided with a response form asking potential participants to either opt-in or opt-out of the study. Nil opt-out responses were received. Furthermore, the mailed-out invitation to participate in research arriving was out of context when the research was to be based in a healthcare setting like the hospital.

### Participant involvement

It was important to weigh up the participant burden in relation to what they got back from their involvement in this research. The altruistic motive of helping others can be a behaviour driver, but if there are obstacles to doing so it may not be enough. Consider (if applicable): the time of available study appointments; the location of the research and ease of getting there; any cost incurred by participating including time off work, travel, and parking; the number of study visits and duration of appointments; and availability of child care. While it is unethical to provide incentives of a disproportional magnitude (financial or otherwise) that may coerce individuals to participate, especially those who are economically vulnerable, or where inducements are undue [14], it is unreasonable not to make participation as easy and rewarding as possible for those who do consent. Reimbursement for time and travel were not deemed feasible for WATCH Study participants. Parking permits were issued to cover all parking expenses associated with study visits and a light meal was provided on the occasions when fasting samples were collected.

### Prioritising ethics approval

The detail in the planning and development phase of the research project is often intertwined with the writing of the ethics application. However this can quickly lead to lapsed judgement about your priorities. In wanting to get started on the research the focus became obtaining approval by the ethics committee. While it is the ethics committee's responsibility is to represent participants and researchers alike, the nature of the application process can hamper achieving the most desirable outcomes for both. Take, for example, the recruiting materials which are described below. By focusing too heavily on the primary function of informing the participant prior to consent we produced a document that was unable to maintain the interest of the reader and may not have been well understood.

## Recruiting materials

### Visual appeal and length of the information statement

The written information provided to potential subjects should reflect the nature of the research, not only in terms of content but also visual presentation. In our case, pregnancy is generally regarded as a positive time in a woman's life, hence we wanted to reinforce this with the written materials we provided in the revised study (copy of WATCH Study Information pamphlet available from author AH on request). The use of images including a study logo, colour and using a pamphlet format has helped us to convey the information we need participants to be aware of in order for them to provide informed consent. While the ABCD recruiting material did include a study logo, it was not colour printed and was presented as formal document rather than pamphlet style.

The length of the text is another important factor that needs to be considered. With careful deliberation we were able to condense what had been two separate information statements totalling just over eight A4 pages in length, into one single double-sided A4 information pamphlet for the WATCH Study. This information pamphlet has been more favourably received by not only by potential participants but also by the other healthcare professionals who see our participants over the course of standard antenatal care.

### Readability of text

The ease of readability of the information statement is too often neglected [15] despite the simplicity in considering this. Readability statistics are available as part of the Microsoft Word (Microsoft Office Word 2003) spelling and grammar functions and they provide an objective measure of how easy materials are to read. The Flesch Reading Ease score rates text on a 100-point scale; the higher the score, the easier it is to understand. Additionally you receive a grading for your text, known as the Flesch-Kincaid Grade Level, which ascribes the text a school grade (United States) level. For example, a score of 8.0 means that an eighth grader should be able to understand the document [16]. Both are calculated using formulas that consider the average sentence length and average number of syllables per word [16]. For most standard documents, the aim is for a Flesch Reading Ease score of approximately 60 to 70 and a Flesch-Kincaid Grade Level of 7.0 to 8.0 [16]. We were able to reduce our Flesch-Kincaid Grade Level from 10.9 for the ABCD Obesity Study and 11.3 for the Math Model recruiting materials, to 7.1 for the combined WATCH study information pamphlet.

### General approach and content

This incorporates many of the factors previously described and will be defined by the level of detail put into developing the study design. Ultimately we had to put ourselves in the position of potential participants and write the study materials from this viewpoint. Ethics committees provide guidance on what information must be given. But it is ultimately the researchers responsibility to ensure that we communicate effectively with potential participants in a manner which aims to encourage participation. From our experience we would recommend seeking advice from those with expertise in recruiting who understand the common mistakes that researchers make when designing the recruitment protocol and materials. Additionally using materials that have proven to be successful as a template may also be advantageous.

### Limitations

Whist we believe that it was the multiple changes that resulted in our improved response rate, the empirical study design is such that we cannot provide direct evidence that all of the changes contributed to the observed improvement. It is possible that only some of the strategies were instrumental in improving the response rate, or even just one. Because all of the changes were made at once we cannot quantify the relative contributions of each. It is even possible that one or more of the changes may have had a negative impact on potential respondents, but that the positive changes compensated so that overall there was still a significant net improvement in our response rate. Future research studies will be required to determine which are the most efficient strategies for ensuring recruitment protocol success.

## Conclusion

This paper describes an attempt that was made to recruit participants into a nutrition-based research study of pregnancy and early childhood, and the knowledge that was gained when this attempt initially failed. The lessons learnt are applicable to those who may try to recruit participants for their own research projects. We hope that by sharing our experience we contribute to the knowledge-base for successful recruitment and help prevent others from making the same simple mistakes. Implementation of effective recruiting strategies will facilitate the achievement of the research objectives without superfluous burden to the study timeframe and resources.

## Competing interests

The author(s) declare that they have no competing interests.

## Authors' contributions

AH carried out the literature review, expert consultation, statistical analysis and drafted the manuscript. RS conceived of the study and assisted with its coordination. CC and RS participated in the overall design of the study and the revision of the draft manuscript. All authors have read and approved the final manuscript.

## Pre-publication history

The pre-publication history for this paper can be accessed here:


